# Holistic management of epilepsy in adults with intellectual development disorders

**DOI:** 10.1002/epd2.70272

**Published:** 2026-05-22

**Authors:** Elena Fonseca, Antonella Riva, Samuel López‐Maza, Malin Zaddach, Marie‐Claire Porter, Ingo Borggraefe, Lance Watkins, Andreas Schulze‐Bonhage, Rohit Shankar, Pasquale Striano, Lina Nashef

**Affiliations:** ^1^ Epilepsy Unit, Department of Neurology Universitat Autònoma de Barcelona, Vall d'Hebron University Hospital, Vall d'Hebron Barcelona Hospital Campus Barcelona Spain; ^2^ Research Group on Status Epilepticus and Acute Seizures, Vall d'Hebron Research Institute (VHIR), Vall d'Hebron University Hospital, Vall d'Hebron Hospital Campus Barcelona Spain; ^3^ Department of Neurosciences, Rehabilitation, Ophthalmology, Genetics, Maternal and Child Health (DINOGMI) University of Genoa Genoa Italy; ^4^ IRCCS Istituto Giannina Gaslini, Full Member of ERN EpiCARE Genoa Italy; ^5^ Division of Pediatric Neurology, Developmental Medicine and Social Pediatrics, Department of Pediatrics Dr. Von Hauner Children's Hospital, University Hospital, Ludwig‐Maximilians‐University Munich Germany; ^6^ The Royal London Hospital, Barts NHS Trust London UK; ^7^ Department of Pediatrics, Division of Pediatric Neurology Comprehensive Epilepsy Center, MUC iSPZ Hauner – Munich University Center for Children with Medical and Developmental Complexity, Dr. von Hauner Children's Hospital Munich Germany; ^8^ Unit for Development in Intellectual and Developmental Disabilities University of South Wales Pontypridd UK; ^9^ Swansea Bay University Health Board, Mental Health and Learning Disability Delivery Unit, Llwyneryr Unit Swansea UK; ^10^ Cornwall Intellectual Disability Equitable Research (CIDER), Peninsula School of Medicine, University of Plymouth Plymouth UK; ^11^ Epilepsy Center, University Medical Center‐University of Freiburg Freiburg Germany; ^12^ Department of Neurology King's College Hospital London UK

**Keywords:** adults, comorbidities, epilepsy, IDD, intellectual development disorders, multidisciplinary care, therapeutic aims

## Abstract

This seminar addresses the complexity of the management of epilepsy in adults with intellectual development disorders (IDD), advocating holistic and multidisciplinary care aligned with the learning objectives of the International League Against Epilepsy. Epilepsy is significantly more prevalent in people with IDD, presenting unique diagnostic, therapeutic, and psychosocial challenges. Accurate diagnosis is hampered by limitations in communication, necessitating reliance on caregivers' observations. In adults with IDD and epilepsy, utilization of diagnostic tools, e.g., video electroencephalograph (EEG) monitoring, is helpful but can be challenging. Management requires careful consideration of cognitive and behavioral comorbidities and the effects of antiseizure medications (ASMs) on cognitive function and quality of life (QoL). Personalized treatment plans should balance seizure control against ASM side effects, prioritizing therapeutic goals aligned with individual patient needs. Effective multidisciplinary care involves primary care physicians, neurologists, psychiatrists, psychologists, social workers, therapists, and specialized nursing staff. Transition periods, including from pediatric to adult care, and from the family home to professional care, represent critical phases requiring structured planning and collaboration among health and social‐care providers, patients, and caregivers. Current gaps in integrated care include the need for targeted therapeutic approaches, more tailored cognitive and behavioral assessment tools, guidelines for managing pregnancy and hormonal considerations, management of commonly associated comorbidities, including non‐epileptic paroxysmal events, and reduction of preventable morbidity and mortality, including sudden death. This seminar emphasizes the necessity of addressing these gaps to advance care standards, promote research, and ultimately improve patient outcomes. The manuscript includes two anonymized illustrative clinical case studies. Practical recommendations include systematic reassessment of underlying etiologies, including genetic counseling and testing, reviewing the electroclinical diagnosis, careful selection and titration of ASMs to minimize cognitive and behavioral impacts, and structured transition. Addressing these challenges and implementing an integrated care model could significantly enhance QoL for adults living with IDD and epilepsy.

**Key points**

- Multidisciplinary collaboration is key to optimizing epilepsy care in adults with intellectual development disorders (IDD), particularly in the presence of comorbidities.
- Treatment plans should be individualized, carefully balancing seizure control with potential adverse effects of medications.
- Well‐structured transition pathways from pediatric to adult care are crucial to ensure continuity and prevent gaps in care.
- Sleep disturbances are common in individuals with IDD and epilepsy, with a bidirectional effect, and must be routinely assessed, as they can significantly impact seizure control.
- Cognitive function in people with IDD and epilepsy requires adapted assessment tools, while behavioral issues demand integrated, targeted interventions.
- Women with IDD and epilepsy face unique challenges during pregnancy and motherhood, underscoring the need for specific clinical guidance.
- Targeted research and tailored clinical guidelines are urgently needed to address unmet needs in cognitive evaluation, sleep management, reproductive health, and management of behaviors that challenge in this population.

**ILAE Curriculum Learning Objectives**

6.1.4 Demonstrate the ability to recognize and manage the special needs of persons with epilepsy and intellectual development disorders

- Re‐investigate if the underlying etiology is unknown and review electroclinical diagnosis, as this may influence management
- Recognize the difficulty in understanding the basis underlying episodic events and confirm their nature with EEG video recording as needed
- Manage frequent comorbidities in a multidisciplinary service
- Anticipate the need for transition and facilitate this at different stages in life
- Manage rescue treatment in the community to reduce excess morbidity and mortality, and minimize disruption for the individual and family

6.1.5 Manage cognitive changes in epilepsy and the cognitive effects of AEDs

- Individualize treatment aims and choices: agree on therapeutic aims in relation to seizure control, safety, and quality of life
- Consider where better control of ongoing epileptic activity, including sleep‐related, in epileptic encephalopathy (EE)/ developmental EE (DEE) may improve cognition and function
- Minimize total drug load to reduce the negative impact on cognition of antiseizure medications.

## 1 INTRODUCTION AND BACKGROUND EPIDEMIOLOGY

Epilepsy is a common neurological disorder with an estimated prevalence of 1% and an annual incidence of approximately 50 new cases per 100 000 in high‐income countries.^1^ Intellectual development disorders (IDD) affect approximately 1.55% of the global population with a range of 1%–3%.^2^, ^3^ The terminology “intellectual development disorders” is based on the 2022 updated International Statistical Classification of Diseases and Related Health Problems (World Health Organization; ICD‐11), with IDD replacing the ICD‐10 terms “mental retardation” and “intellectual disability”. This has been implemented by the American Psychiatric Association via the Diagnostic and Statistical Manual of Mental Disorders, 5th Edition (DSM‐5 criteria), which also replaces “mental retardation”.

In accordance with the DSM‐5 criteria, IDD must include deficits in both intellectual and adaptive functioning, crucially with the onset of disability during the developmental period. The severity of IDD can range from ‘mild’ to ‘profound’, requiring complete dependence on caregivers (Table 1). Epilepsy in individuals with IDD typically has an earlier onset, shows higher rates of treatment resistance, and is frequently accompanied by multiple comorbidities. Its prevalence is higher than in the general population, with a pooled estimate of 22.2% (95% confidence interval [CI] 19.6–25.0)^1^ across all levels of IDD, and a clear correlation between epilepsy prevalence and the severity of IDD. For those with mild IDD, who constitute the majority, the epilepsy prevalence is ~10%, compared with ~15% with moderate IDD, ~30% with severe IDD, and ~50% with profound IDD.^1^ Comorbidities, including motor, speech, behavioral, cognitive, psychiatric, and sleep‐related issues, as well as systemic comorbidities, including gastrointestinal disorders, are common in patients with IDD and epilepsy, and may develop over time.^5^, ^6^, ^7^, ^8^, ^9^ Comorbidities may be linked to specific developmental and epileptic encephalopathies (DEEs), which have their own needs at different stages, such as gait disturbance in adults with Dravet syndrome.^9^ Comorbidities may also be linked to the long‐term use of antiseizure medications (ASMs).^5^ Around 70% of people with IDD and epilepsy are considered to be pharmacoresistant, requiring multiple ASMs^10^ with an increased side‐effect burden and risk of sudden death in epilepsy (SUDEP) compared with those without IDD,^11^ and reliance on multiple aspects of local healthcare systems throughout their lives.

**TABLE 1:** Definitions of severity categories for IDD.

Severity category	DSM‐5 criteria (severity classified on the basis of daily skills)^4^	AAIDD criteria (severity classified on the basis of intensity of support needed)^4^
Mild	Able to live independently with minimum levels of support. (Previous DSM‐IV IQ range: 50–69)*	Intermittent support needed during transitions or periods of uncertainty
Moderate	Independent living may be achieved with moderate levels of support, such as those available in group homes. (Previous DSM‐IV IQ range: 36–49)*	Limited support needed in daily situations
Severe	Requires daily assistance with self‐care activities and safety supervision. (Previous DSM‐IV IQ range: 20–35)*	Extensive support needed for daily activities
Profound	Requires 24‐h care. (Previous DSM‐IV IQ range: <20)*	Pervasive support needed for every aspect of daily routines

*Note*: Adapted from Committee to Evaluate the Supplemental Security Income Disability Program for Children with Mental Disorders, 2015.

Abbreviations: AAIDD, American Association on Intellectual and Developmental Disabilities; DEE, developmental and epileptic encephalopathy; DSM‐5, Diagnostic and Statistical Manual of Mental Disorders, 5th Edition; IDD, intellectual development disorders; IQ, intellectual quotient.

* DSM‐IV severity categories based on IQ scores only.

**FIGURE 1:**
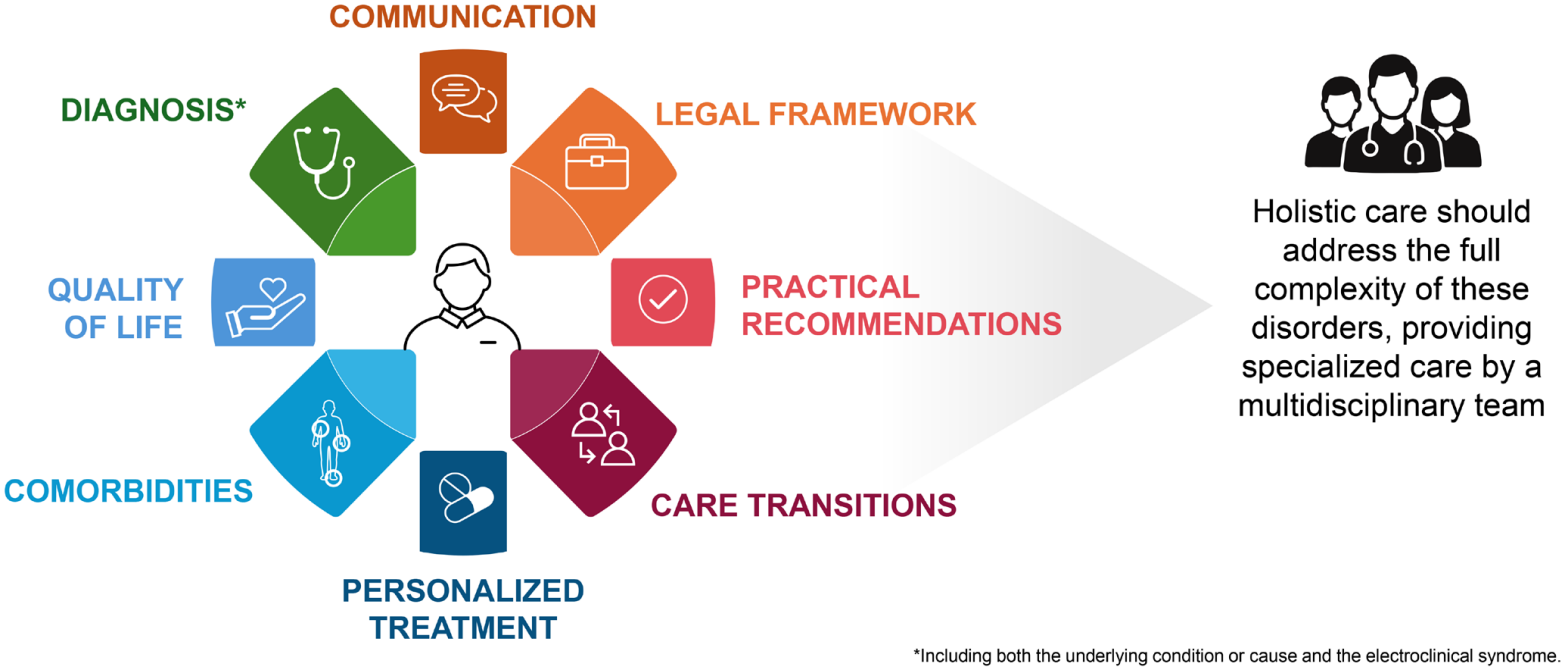
Holistic considerations for the management of adults with intellectual development disorders and epilepsy.

**FIGURE 2:**
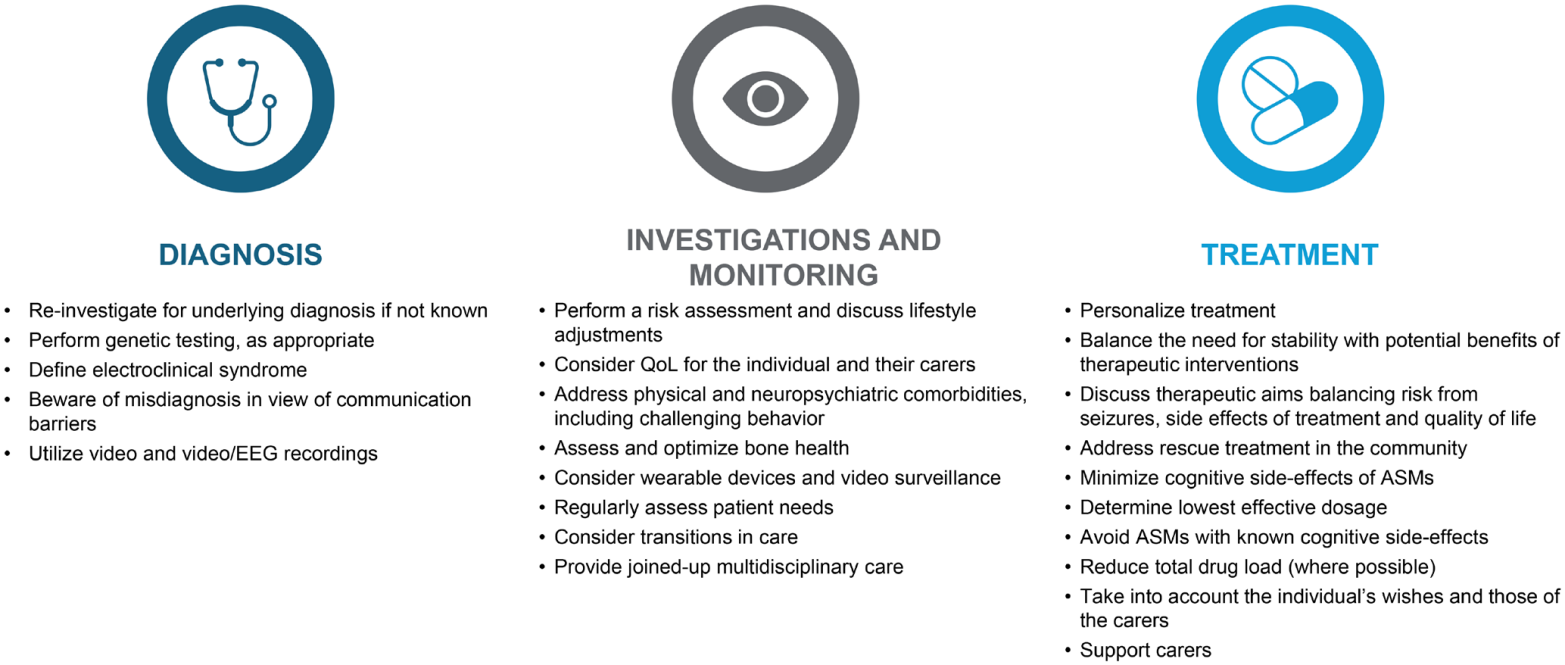
Considerations in the clinical journey of an adult patient with intellectual development disorders and epilepsy. ASM, antiseizure medication; EEG, electroencephalograph; QoL, quality of life.

Among holistic considerations for the management and care provision of adults with IDD and epilepsy (Figure 1), there are established areas of need that require improvement.^12^, ^13^ While multidisciplinary care is integral to pediatric management, this is not always the case for adults with IDD and epilepsy, where care may be disjointed. There is variability in the care provided and a lack of internationally accepted standards, as well as challenges in engagement.^14^, ^15^ This seminar manuscript was developed following a workshop of epileptologists. It draws on themes discussed during the workshop and incorporates findings from an independent, targeted, non‐systematic literature review on epilepsy in adults with IDD, with a focus on holistic management in this population. This seminar addresses diagnosis, misdiagnosis, and treatment of epilepsy, and highlights comorbidities (Figure 2). It focuses on the ability to recognize and manage the needs of adults with IDD and epilepsy, including DEEs, as well as address optimal broader management of epilepsy, including the often‐overlooked cognitive effects of ASMs, both learning objectives in the ILAE curriculum.^16^ Challenges in management, gaps in knowledge, and disparities in care are also considered. While the seminar adopts a holistic perspective, its primary focus remains on clinical management in relation to epilepsy, with broader medical management and determinants of well‐being acknowledged but not explored in detail.

## 2 METHODOLOGY

This seminar manuscript originated following a dedicated multinational workshop of epileptologists, organized by Jazz Pharmaceuticals in May 2024, which focused on clinical, diagnostic, and organizational challenges in providing holistic care for adults with DEE, and more broadly IDD and epilepsy. Following the workshop, and independent from it, the authors decided to co‐author a seminar manuscript on the subject of holistic management of epilepsy in adults with IDD, informed by the experience of the participants, the materials discussed at the meeting, and an independent, targeted, non‐systematic literature review. Literature searches focused on adults with epilepsy and IDD, and included epidemiology, diagnostic pathways, comorbidities, service provision, and therapeutic strategies. The aim was to provide a clinically grounded and educational synthesis rather than a systematic or exhaustive review. The manuscript also includes two anonymized illustrative clinical case studies (see Appendix A). Informed consent was obtained for both cases from parents or legally authorized representatives.

## 3 CHALLENGES IN SERVICE PROVISION

There is global variation in the availability and quality of healthcare for people with epilepsy in general, with no comparative studies. Higher‐income countries have more specialized clinics and services. However, significant challenges exist due to a lack of joined‐up care.^17^ Studies from Australia and the UK show higher rates of hospital readmissions and emergency department visits for seizures, aspiration pneumonia, falls, injuries, and constipation in people with IDD with epilepsy compared with those without IDD with epilepsy, often because of challenges in managing comorbid conditions.^18^, ^19^, ^20^, ^21^, ^22^ Admission due to seizures was the most common cause in the general population of patients with epilepsy.^19^ However, the presence of multiple comorbidities, including respiratory difficulties, motor impairment, and neuropsychiatric diagnoses, further increases the risk of admission in people with IDD and epilepsy.^23^, ^24^, ^25^ An online qualitative international survey, conducted by the International Bureau of Epilepsy, highlighted the negative and pervasive burden, both caregiving and financial, faced by 48 families supporting a family member with IDD and epilepsy.^26^ Moreover, social‐care costs are reported to exceed healthcare costs in this population, particularly among those with severe IDD and those with refractory seizures.^27^

Significant challenges are recognized at patient, clinical, and service level with variation in service availability, resources, training, and delivery.^14^ Based on some of the authors' experiences of neurology/epilepsy clinics in hospitals in the UK, varying expertise, limited capacity, appointments scheduled too early or late in the day, crowded waiting rooms, delays in being seen, small clinic rooms, and insufficient time, against a background of high staff turnover including among carers, all conspire against good care. Moreover, where people with IDD and epilepsy require admission to acute hospitals, facilities can be suboptimal, often with insufficient reasonable adjustments made, although a legal requirement. For example, carers may not be able to stay overnight when needed. Whilst facilities for medium‐term admissions in specialized units are scarce, in the UK, as in many other countries, the management of people with IDD and epilepsy is often in secondary or tertiary care. Outpatient clinics may be held in hospitals away from community‐based psychiatric/intellectual disabilities services, hampering the ability to provide joined‐up care.^13^, ^14^, ^17^, ^28^ In lower‐income settings, access to diagnostic tools and treatments is limited.^29^ Stigma and cultural beliefs regarding seizures and IDD may also limit care and perpetuate inequalities,^30^, ^31^ while close family support may enhance safety and quality of life (QoL).^32^, ^33^

### 3.1 Transition

Transition of care occurs at more than one stage in the life of those with IDD and epilepsy. Pediatric to adult transition of medical care is widely discussed as a time when continuity of care can be disrupted, risking deterioration in seizure control and higher hospitalization rates.^34^, ^35^ Transition requires early planning, overcoming communication barriers, and ensuring continuity of care. There are ongoing difficulties related to the successful realization of this transition,^36^, ^37^ such as late planning, lack of provider expertise, and difficulties in addressing the needs of individuals with IDDs in adult care.^36^, ^38^, ^39^ Pediatric to adult transition clinics exist, but there is variability in the availability of this service for those with epilepsy, with and without a diagnosis of IDD.^39^, ^40^ Addressing these barriers through structured programs would enhance continuity of care and is more likely to guarantee continuous, consistent communication between pediatric and adult services. Transition also occurs at other stages. For some, whether at home or in residential care/supported living, their condition may progress such that their needs are no longer met by their care setting. For others, living at home with their parents, transition again looms as parents age. Parents can be left to navigate this alone, battling social services with no roadmap or formal process of anticipation and support, nor guidance or standards to follow. More work is needed to define standards of care in this regard to facilitate transition for the individual concerned.^41^

### 3.2 Determinants of quality of care

Clinicians are increasingly aware of the need to address QoL for people with epilepsy in everyday clinical practice in different patient groups and clinical trials and post‐surgery.^42^ Available outcome measures, however, may be limited to evaluate QoL in people with IDD.^43^ There is a need to define QoL priorities for adults with IDD and epilepsy across different patient groups.^43^ The extensive Quality of Life in Epilepsy Inventory (QOLIE‐31), for example, is reportedly superior to the EuroQoL 5‐dimension, 3‐levels (EQ‐5D‐3L) in evaluating QoL in severely affected patients with epilepsy.^44^ Seizure freedom and minimal side effects of ASMs are considered important determinants of QoL in epilepsy in general^44^; however, in drug‐resistant epilepsy, other measures can also be important. In recent years, clinical vignettes have been used to assess QoL determinants.^45^ Other measures considered include predictability and seizure‐free days.^46^

### 3.3 Capacity and decision‐making

The legal framework for medical decision‐making in people with IDD varies in different countries. People with IDD need to be empowered to participate as fully as possible in any decision affecting them. Article 12 of the United Nations Convention on the Rights of Persons with Disabilities establishes that people with disabilities have the right to legal capacity on an equal basis with others in all aspects of life. This framework calls for supported decision‐making to replace substituted decision‐making and for the person's preferences to be considered first with the necessary support provided.^47^ In clinical practice, capacity should be assessed in relation to the specific decision under consideration, recognizing that many individuals may be able to express preferences when information is presented in an accessible format and when communication is facilitated by caregivers or familiar professionals.^48^ National regulations on consent and safeguarding guide clinicians in determining when proxy or legally appointed representatives must be involved.

Beyond the legal dimension, social determinants play a substantial role in shaping access to care and health outcomes.^49^, ^50^ Limitations in education, employment, autonomy, and community resources can influence engagement with services, continuity of follow‐up, and adherence to treatment plans. Stigma and reduced social participation may further compound these barriers.^51^ Awareness of these contextual factors helps clinicians tailor recommendations that are realistic and sustainable, ensuring that medical decisions are not made in isolation from the person's lived environment and support network.

### 3.4 Accidental injury, bone health, and excess mortality (including SUDEP)

Reported accidental injury rates in people with IDD and epilepsy vary. Reduced opportunities to engage in daily activities^34^ may lower the risk in people with more severe disabilities, while accidents resulting from daily activities (e.g., cooking) are common in people with epilepsy and those with milder IDD.^52^, ^53^, ^54^, ^55^ Contributing factors include the impact of ASMs and reduced mobility.^56^ The incidence of both osteoporosis and bone fractures is high in people with IDD and epilepsy. In a study of institutionalized adults with IDD and epilepsy, 40% had at least one clinical fracture during 7 years of follow‐up.^57^ Assessing and optimizing bone health is essential in this patient group and is not routinely addressed.

A systematic review of 16 studies found that epilepsy in people with IDD was associated with increased mortality compared with the general population.^58^ In one study from the UK, the risk of death in people with IDD and epilepsy was 3.2 times greater than the general population for men (95% CI: 2.7–3.8) and 5.6 times greater for women (95% CI: 4.6–6.7).^59^ In another study, the risk of death was 16.8 times greater (95% CI: 10.7–26.5) for those having more than weekly seizures versus those having less than weekly seizures.^60^ Studies also report an increased overall risk of SUDEP in people with IDD compared with those without^11^ and with specific underlying genetic conditions.^61^ Several large cohorts, including register‐based data sets, have shown that comorbid epilepsy and IDD significantly raise mortality risk, often due to refractory seizures, impaired mobility, aspiration risk, and issues with coordination of care.^52^, ^62^, ^63^ A UK‐based study of mortality in IDD concluded that pervasive health inequalities and missed prevention opportunities may be a factor in the earlier epilepsy‐related mortality observed in IDD.^64^ National and international guidelines stress the importance of SUDEP communication at the earliest opportunity. There remains clinician‐mediated bias on when and with whom SUDEP discussions should take place.^65^, ^66^ The SUDEP and Seizure Safety Checklist^67^ consists of known modifiable and non‐modifiable risk factors associated with SUDEP and other harm, for use in a structured risk assessment and person‐centered discussion,^68^, ^69^, ^70^, ^71^ leading to a personalized risk profile. It has been shown to influence behavioral change, thereby reducing modifiable risk factors, with the most impact in those considered at highest risk.^72^, ^73^ Documentation of SUDEP discussion and periodic reassessment is needed.

## 4 EPILEPSY MANAGEMENT IN PEOPLE WITH IDD

The clinical management of epilepsy in people with IDD is hampered by many factors that can have an impact on care, including diagnostic challenges, difficulties with monitoring events, symptoms, and comorbidities, as well as assessment of ASM adverse effects and drug–drug interactions. These factors may be negatively impacted by communication barriers^74^ and by the framework of care provision needed to support those with combined IDD and epilepsy, involving patients, families, legal guardians, and professional caregivers. Communication barriers may arise from a combination of limited receptive and expressive language, reliance on carers to interpret information about symptoms, seizures, ASMs, and comorbid conditions, as well as issues with trust, credibility, and consistency of information provision.^74^ These challenges can be addressed by appropriate education and counseling using accessible language and other delivery formats involving the family and carers. Caregiver‐frameworks underpinned by evidence relating to carer burden, care pathways, patient information needs, and shared decision‐making structures can be useful for overcoming communication barriers.^26^, ^75^

### 4.1 Diagnostic challenges

#### 4.1.1 Etiological and electroclinical diagnosis

In people with IDD and epilepsy, regularly reconsidering the etiology and re‐investigating for an underlying diagnosis may be needed.^76^ Genetic advances, in particular whole‐genome sequencing and microarrays, have greatly advanced the ability to identify the genetic cause in many cases,^77^ which can have an impact on prognosis and choice of treatment, as well as inform about systemic associated comorbidities. There are many different underlying genetic etiologies in epilepsy in the context of IDD. These include mechanistic domains resulting in malformations of cortical development or alterations in synaptic function and metabolic transport pathways.^78^ For example, *SCN1A*‐, *STXBP1*‐, and *CDKL5*‐related encephalopathies have all been associated with combined, severe, early‐onset epilepsy and IDD or developmental impairment.^79^, ^80^, ^81^ Pathogenic variants of *SCN1A* are also the primary genetic cause of Dravet syndrome, a DEE associated with cognitive impairment.^82^ Reaching an etiological (often genetic) diagnosis is increasingly important. Ongoing research may enable more personalized therapy effective against both cognition and seizures, with gene‐ or mechanism‐targeted new therapies (see Section 9).^83^

Some adult patients with epilepsy and IDD, for example, may satisfy criteria for Lennox–Gastaut syndrome, but may not have had that diagnosis previously made, having been diagnosed with ‘symptomatic generalized epilepsy’ according to the 1989 ILAE criteria.^84^ Reviewing early history, seizure types, historical and current awake and sleep electroencephalographies (EEGs), and any prolonged sleep recordings is necessary to clarify the electroclinical diagnosis, potentially leading to more treatment options.

#### 4.1.2 Classifying and monitoring episodic events

A systematic review exploring the misdiagnosis of epilepsy in people with IDD reported that up to 38% of people with IDD were misdiagnosed, with misinterpretation of behavioral, physiological, syndrome‐related, medication‐related, or psychological events.^85^ Understanding the basis for episodic events is essential for appropriate management. The diagnosis of epilepsy in general is based on expert assessment, including an accurate history from both the patient and a witness. People with IDD and epilepsy can find it challenging to report semiological elements of their seizures, including auras or postictal impairments, while information from observers may be compromised by a lack of consistency and misinterpreted observations. This poses difficulty in differentiating epileptic seizures from non‐epileptic events, including behavioral stereotypes or psychogenic events. There is a risk of misdiagnosis or diagnostic overshadowing by healthcare professionals, who may under‐ or overestimate the occurrence of epileptic seizures. Video EEG is often a prerequisite for classification of events to confirm if they are epileptic or not and reach an electroclinical syndromic diagnosis, essential to guide treatment. Video‐EEG monitoring was reported to change the classification of events in more than one third of patients with IDD and epilepsy (36.5%).^86^ Inpatient monitoring may be difficult because of behavioral challenges. Home videos and the less disruptive prolonged home EEG video recordings, where available, can be very helpful.^87^, ^88^

Developing technologies may also prove helpful.^89^ Subcutaneous EEG recordings, for example, can allow for almost continuous EEG recordings for months to years, though exploration of their potential use remains preliminary in individuals with IDD and epilepsy.^90^ Wearable devices^91^, ^92^, ^93^, ^94^, ^95^ and AI‐supported video surveillance systems^96^ can provide additional information on seizure frequency, as well as severity, to guide treatment. By providing objective outcome measures, such devices may also facilitate the inclusion of people with IDD and epilepsy in clinical research for antiseizure treatments, where they are underrepresented.^90^, ^97^, ^98^

#### 4.1.3 Non‐epileptic paroxysmal events

There was no evidence in one study of diagnostic delay when differentiating psychogenic from epileptic seizures in people with mild IDD compared with those with normal cognition.^99^ Studies report different rates of non‐epileptic paroxysmal events in people with IDD.^100^, ^101^ Such events in people with IDD present a significant challenge, with a growing recognition that they should not be approached through the same lens as functional or dissociative seizures.^100^, ^101^ Non‐epileptic paroxysmal events in individuals with IDD are likely to be of a complex, multifactorial nature linked to a range of additional neurological, developmental, and environmental factors.^101^ A person, unable to express distress or discomfort in other ways, might exhibit seizure‐like behaviors in response to physical or emotional triggers.^101^ In this context, these events require a comprehensive individualized approach to assessment and management. This should start with the recognition that people with IDD and epilepsy often have multiple causes for paroxysmal events and that labelling these events as epileptic seizures is common.^102^ Assessment of potential non‐epileptic paroxysmal events should include a thorough systemic and neurological evaluation, assessment of sensory and communication needs, and consideration of the individual's psychological and environmental context.^103^ Treatment should explore combining neurological and medical care, sensory modulation, communication strategies, and behavioral interventions in a multidisciplinary approach.^101^

### 4.2 Antiseizure medications

Prescribing ASMs for people with IDD and epilepsy is hampered by limited evidence from clinical trials. A traffic light system was proposed in the UK in 2017, under the auspices of the Royal College of Psychiatrists, to guide ASM prescribing in this setting based on both the strength of evidence and utility.^104^ Under this system, which has been updated and extended since its introduction,^105^, ^106^ existing ASMs are classified in three categories: ‘red’ (only to be used in exceptional circumstances), ‘amber’ (could be considered if benefits outweigh risks or as second‐line treatment), or ‘green’ (considered as first‐line treatment).

#### 4.2.1 ASMs in IDD and behavioral challenges

People with IDD and epilepsy have higher levels of comorbidities compared with people without IDD. Comorbidities include physical, neurodevelopmental and neuropsychiatric disorders, which can include autism spectrum disorder and attention‐deficit hyperactivity disorder.^5^, ^6^, ^24^, ^34^, ^107^, ^108^, ^109^, ^110^ Older adults (over 40 years) with IDD and epilepsy often exhibit further multi‐morbidities.^107^ They experience polypharmacy, particularly with antipsychotics, which can negatively affect QoL.^111^ In institutionalized or supported‐living settings, they may exhibit fewer seizures than younger individuals, likely due to more regimented medication administration.^107^, ^111^ In a systematic review, more severe epilepsy was associated with neuropsychiatric comorbidity, as was the presence of IDD when compared with the general population, adding to the complexity of managing epilepsy in these individuals.^112^ One study described the frequency of challenging behaviors in adults with both IDD and epilepsy at a tertiary epilepsy center, with self‐injurious behavior presenting in 35% of subjects, stereotyped behavior in 60%, and aggressive/destructive behavior in 63%.^113^ A systematic review and meta‐analysis looking at epilepsy and challenging behavior in adults with IDD reported contradictory findings.^114^ There was no significant association between epilepsy and challenging behavior, but after sensitivity analysis of ten studies, a significantly higher rate of overall challenging behavior was found in the epilepsy group (effect size: 0.16) compared with the non‐epilepsy group. Aggression and self‐injurious behavior, but not stereotypy, both showed a significantly higher rate in the epilepsy group, with very small effect sizes (0.16 and 0.28, respectively).^114^ Behaviors that challenge, defined as culturally abnormal behaviors of an intensity, frequency, or duration that jeopardize the safety of the individual or others, also remain a significant management issue in this setting.^115^ Any worsening of behavior, which can be medication‐related but could have diverse causes, risks the individual's QoL, residential placement or daycare facility, and needs to be avoided and/or addressed early. A best practice checklist has been suggested to provide clinicians with a structured framework to consider causal explanations for behaviors that challenge in this population.^115^, ^116^ The checklist includes: biological (history taking, clinical examination, and physical investigations), mental health/psychological/psychiatric/behavioral, social and medication factors, all of which may help with their assessment and management of these behaviors.^115^ The 2016 report of the Intellectual Disability Task Force of the Neuropsychiatric Commission of the International League Against Epilepsy (ILAE) proposed a range of research and clinical practice recommendations, including that any child or adult with IDD, epilepsy, and behavioral disorder should be provided with multidisciplinary care.^6^ Behavioral problems may lead to choosing ASMs with sedating properties or to administering sedating medications, which carry inherent risks.^117^

There are limited sufficiently powered studies to guide evidence‐based ASM prescribing in people with IDD and epilepsy.^110^, ^118^ People with IDD and epilepsy are often excluded from or at least underrepresented in clinical trials, partly due to complexities around consent and the heterogeneity in their cognitive and adaptive functioning.^98^, ^119^ In many countries, ethics committees require justification for the inclusion of people with IDD in clinical trials, the consequence of which is a lack of data for modern treatments and associated risks.^119^ While recognizing ethical and practical considerations involved, the counterargument is that there needs to be good justification for excluding special groups from clinical trials, particularly for conditions which commonly affect them.

While some studies suggest responses to certain ASMs may be similar in those with and without IDD,^120^, ^121^ this has to be reconciled with a higher incidence of refractory epilepsy in the subgroup of people with IDD and DEE.^122^, ^123^ Furthermore, in DEEs, multiple seizure types are often present, necessitating an awareness of the efficacy of ASMs in different seizure types, as well as the potential for seizure exacerbation with narrow‐spectrum ASMs.^124^ Barriers to adherence to treatment are not well studied in this group and deserve more focus. Moreover, tolerability and side effect profiles often demand more careful monitoring due to limitations in self‐reporting. Thus, beyond seizure control, treatment in those with IDD and epilepsy requires awareness of the often‐atypical manifestations of adverse treatment effects, including behavioral and cognitive effects, which are discussed further below.

#### 4.2.2 ASMs and cognition

Cognitive impairment is associated with both underlying pathology, seizure load/epileptiform discharges, and ASM side effects.^125^, ^126^ Epilepsy specific parameters such as seizure frequency (and ongoing epileptic activity) may contribute more to cognitive impairment in epileptic encephalopathies. Epilepsy onset in such patients is often associated with developmental slowing/regression. This raises the question that ASM treatment might improve cognitive functioning.^127^ Those with treatment‐resistant epilepsy, however, often receive ASM polytherapy without achieving seizure freedom in the majority. Yet polytherapy is associated with a higher risk of adverse effects on cognition.^128^ Moreover, ASMs differ in their effect on cognition, with negative effects associated with treatment with phenobarbital, phenytoin, topiramate, and carbamazepine; whereas levetiracetam and lamotrigine have been considered to have the least impact on cognition.^129^, ^130^, ^131^ A narrative review concluded that in DEEs, cannabidiol (CBD), fenfluramine, levetiracetam, brivaracetam, and lamotrigine may have positive effects on some aspects of cognition.^132^

Cognitive improvement might be achieved after epilepsy surgery in a small proportion of suitable patients.^133^, ^134^ There is sparse evidence that treatment of EE with standard ASMs leads to significant cognitive improvement.^129^ Regarding newer agents, CBD led to clinical improvement in selective attention and on a caregiver‐rated behavioral measure in a patient cohort of drug‐resistant epilepsy comprising both DEE and EE.^135^ In patients with Dravet syndrome, fenfluramine improved executive function, also suggesting an effect beyond seizure reduction.^136^ In DEE, emerging precision‐based treatments, discussed further below, aim to target the underlying defect, thus addressing the spectrum of disease‐specific symptoms comprising seizures, cognitive impairment, and other comorbidities. These approaches align with the concept of disease modification as not ‘only’ seizure control.

Treating clinicians need to consider the following to minimize cognitive and other side effects of ASMs:

- Discuss/define therapeutic aims for the individual concerned, namely reduction or freedom from disabling seizures, with the patient/caregivers. A range of possible therapeutic aims are discussed further in the next section.
- Slowly titrate to the lowest effective dosage and choose the agent with the most favorable pharmaceutical profile for the individual. If the last incremental increases have had no added value, slowly titrate back to the previous effective dose.
- Avoid, where possible, ASMs with known cognitive side effects.
- Apply increased caution for those with a history of neuropsychiatric problems/mental illness and identify any emerging behavioral problems early. These may relate to speed of titration and dose as well as overall pharmacological load.
- Wherever possible and considered safe to do so, within agreed therapeutic aims, reduce the total number of ASMs as well as total drug load.

### 4.3 Therapeutic aims in relation to seizure control in people with IDD

While seizure freedom/control in epilepsy is desirable, as discussed above, it should not be at the expense of unacceptable side effects from ASMs. Brief auras, mild brief focal seizures, or absences, if they do not carry a physical risk, may not affect QoL for some, and decisions about treatment need to consider the person's wishes, needs, activities, and circumstances. The clinician, patient, and carer need to consider together which therapeutic aims are appropriate and achievable with minimum side effects in each case, and how these, if achieved, would enhance safety, reduce morbidity and mortality (including SUDEP risk), and improve QoL for the individual as well as their carers. Various levels in connection with therapeutic aims in relation to seizure control, other than seizure freedom, include:

- Freedom from all but the most minor seizures without physical risk or postictal after effects.
- Freedom from seizures with significant loss of awareness.
- Reducing the need for 24‐h observation for ongoing seizures and the need for rescue medication for seizures.
- Preventing all seizures with a risk of injury (those associated with falls, wandering, or other risk‐associated behavior).
- Increasing seizure‐free days.
- Preventing major disruption and risk associated with the need for emergency care (paramedics, A&E attendance, and/or admission) for status epilepticus or seizure clusters.
- Preventing nocturnal convulsions, thus reducing the risk of SUDEP.

If control of more severe seizures cannot be achieved as outlined above, enhanced monitoring and seizure detection need to be considered to minimize risk and allow for prompt rescue treatment initially in the community. Practice clinical guidelines regarding seizure detection using wearable devices have been published by the ILAE.^89^ Table 2 lists some available devices. At the time of writing, there were two wearable devices commercially available specifically designed for people with IDD and epilepsy: (1) Nightwatch, a photoplethysmographic heart rate module and accelerometer that has shown high sensitivity for major nocturnal seizures in adults and children with IDD and epilepsy living in residential care,^94^, ^95^, ^139^ and (2) Nelli, an AI‐based automated detection system capturing audio and visual data (typically during sleep) that has been shown to capture seizures missed by carers of patients with IDD and epilepsy.^96^, ^146^ More devices are likely to become available in the future.

**TABLE 2:** Seizure detection devices that demonstrate positive future potential.^137^, ^138^

Device	Type	Description
Nightwatch^94^, ^95^, ^139^	Wearable	Photoplethysmographic heart rate module and accelerometer
Epi‐Care^140^, ^141^	Wearable	Accelerometer for detecting GTC seizures
Embrace 1&2 and now EpiMonitor (Empatica)	Wearable	Multimodal^142^—Accelerometer, gyroscope, photoplethysmography, electrodermal activity, and temperature
Seizure Link	Wearable	Surface EMG^143^
UNEEG^144^	Implant (unilateral subcutaneous)	Remote EEG, prolonged monitoring analysis aided by an AI algorithm
Epiminder^145^	Implant (bilateral subcutaneous)	Remote EEG, prolonged monitoring analysis aided by an AI algorithm
Nelli^96^, ^146^	Non‐wearable	Automated detection system capturing audio and visual data (typically in sleep or at rest). AI‐based algorithm

Abbreviations: AI, artificial intelligence; EEG, electroencephalogram; EMG, electromyography; GTC, generalized tonic–clonic.

Most commonly, rescue treatment is with the use of benzodiazepines, along with individualized protocols, which must take into account the individual's seizure duration, frequency and pattern, as well as the caregiver's capabilities.^147^ The choice between nasal or buccal midazolam or other options must consider factors such as availability, ease of administration, speed of action, and patient/carer preference to ensure timely and effective intervention during seizure emergencies. It is also important to consider how often the protocol would result in the use of rescue treatment and adjust this to avoid dependence and reduced efficacy.^147^ Rescue treatment is generally not needed in adults with IDD where there is no history of prolonged seizures or seizure clusters. Furthermore, overuse/misuse of rescue medication can occur and needs to be avoided.^148^ Best practice guidelines have been developed in the UK by the Epilepsy Specialist Nurses Association.^149^, ^150^ An individualized emergency management plan should include descriptions of seizures and seizure duration, or definition of seizure cluster requiring rescue treatment, drug to be administered, dose and route, as well as when to call emergency services.^151^ It should be reviewed on a regular basis.

### 4.4 Epilepsy surgery and neuromodulation

Surgical and neuromodulatory treatments may represent valuable options for selected adults with IDD and epilepsy when conventional pharmacotherapy has proven insufficient. Where patients meet the ILAE definition of pharmacoresistant epilepsy, referral to a specialist or tertiary epilepsy center for comprehensive re‐evaluation is recommended, including formal consideration of epilepsy surgery and neuromodulation.^152^ Procedures such as resective surgery or laser ablation in the presence of focal epileptogenic networks, disconnection procedures, vagus nerve stimulation (VNS), or responsive neurostimulation (RNS) can offer meaningful seizure reduction and, in some cases, improvements in alertness, behavior, and overall functioning.^153^, ^154^, ^155^ Literature on surgical outcomes in patients with treatment‐resistant epilepsy and IDD is mixed in relation to whether IQ affects outcome.^62^, ^156^

Presurgical evaluation presents unique challenges in this population.^157^ Behavioral difficulties, patient tolerance of scan duration, movement artifacts during imaging, limited cooperation with prolonged video‐EEG monitoring, intolerance of invasive diagnostic procedures, and difficulties in completing advanced neuropsychological assessment may restrict assessment, and thus eligibility for surgery.^157^ Neuroimaging acquisition may require sedation/anesthesia, and diffuse and multifocal structural abnormalities may complicate localization and surgical planning.^157^, ^158^ Furthermore, assessing postoperative risks and predicting functional outcomes is more complex in individuals with baseline cognitive impairment.^157^

Despite these barriers, early referral to a comprehensive epilepsy center is essential. Where definitive resective surgery is not possible, neuromodulatory approaches including VNS and other neuromodulatory approaches may offer clinically relevant benefits with a favorable risk profile.^28^, ^154^, ^157^ VNS may have a positive effect on mood without negative cognitive effects associated with ASMs but currently carries some long‐term limitations regarding imaging and may exacerbate sleep apnea. Recommendations should be made within a multidisciplinary framework involving neurologists/neurophysiologists, neurosurgeons and neuropsychologists, with therapeutic options presented to caregivers and, whenever possible, the patient and supported by specialist nurses.^28^ A realistic appraisal of expectations, caregiver capacity, and long‐term support is crucial to ensure that any intervention aligns with the individual's needs, safety, and QoL.^28^

## 5 SLEEP DISORDERS

Sleep is a key factor in the QoL of patients with DEEs,^8^ although studies are limited in adults with IDD and epilepsy in general, and further research is needed. There is a bidirectional relationship between epilepsy and sleep: nocturnal seizures disrupt sleep architecture, and a lack of sleep can aggravate seizure frequency.^159^, ^160^ In some DEEs, such as Dravet syndrome, sleep disorders are reported more frequently than in the general population and general epilepsy cohorts,^8^ challenging seizure control and affecting the developmental outcome and QoL for patients and carers. Sleep disorders include insomnia, frequent arousals, sleep apnea, and daytime sleepiness, which can be aggravated by disrupted sleep due to frequent sleep‐related epileptiform discharges/epileptic seizures,^161^ polypharmacy, and/or the underlying condition. Some ASMs, especially benzodiazepines, can cause respiratory depression, while others can alter sleep quality.^147^, ^162^ Psychiatric problems and environmental triggers can exacerbate sleep problems. In turn, disturbed sleep can affect presentation, behavior, and functioning in the days that follow. More research is needed.

## 6 GENDER, SEXUALITY, AND HORMONAL ASPECTS

The natural course of epilepsy may be different according to sex.^163^ Indeed, pooled estimates have reported the prevalence of epilepsy as being slightly higher in males versus females (24.8% [95% CI: 19.6–30.8] versus 22.2% [17.3–28.1]),^1^ whilst standardized mortality ratios have been reported to be higher in females than males (5.6 and 3.2, respectively),^59^ reflecting the higher baseline mortality in the general population in males. Sexuality, in relation to sex education, sentimental relationships, and gender identification, is often forgotten in people with IDD^164^ and, while there is overlap with the general population with epilepsy, specific studies on sexuality, gender, or hormonal aspects in IDD are lacking. In women with epilepsy in general, reproductive endocrine disorders include anovulatory menstrual cycles, oligomenorrhea, and polycystic ovary syndrome (PCOS), the incidence of which is higher compared with the general population.^165^ Furthermore, cyclic hormonal changes can have a significant impact on seizure control. Catamenial exacerbation of seizures is influenced by fluctuations in neurosteroid levels.^166^ Progesterone, a natural anticonvulsant, can counteract the excitatory effects of glutamate, while estrogens exert proconvulsant properties. Managing menstruation may also be harder for individuals with IDD compared with those without.^167^ Evidence relating to the effectiveness of different contraceptive strategies for individuals with epilepsy and IDD is scarce. A 2023 population‐based US Medicaid analysis of women with IDD found that they were less likely to receive intrauterine devices (IUDs) than women without and were more likely to receive injectable contraception.^168^ Another study in the US reported that neurologists tend to recommend longer‐term methods (implants, IUDs, and injectable contraceptives) for women with epilepsy and IDD,^169^ largely to reduce pregnancy risk given teratogenic ASMs (such as valproate) and adherence concerns. In both men and women in general, sexual dysfunction may be associated with ASMs,^170^ which affect sex hormone levels and the effectiveness of hormonal contraceptives. Some ASMs, such as carbamazepine, phenytoin, and phenobarbital, reduce sex steroid concentrations and alter the hypothalamic–pituitary–adrenal axis,^170^ contributing to fertility problems, and valproate has been associated with PCOS.^171^ Valproate has also been reported with reduced fertility in animal studies, and there are case reports of reversible infertility in male patients,^170^, ^172^ although a recent large epidemiological study did not show a significant association of male infertility with valproate.^173^ In people with IDD and epilepsy, the approach to sexuality and reproductive health needs to be individualized according to the cognitive and decision‐making abilities of these patients. A functional, decision‐specific capacity assessment, focusing on the person's ability to understand, use, and communicate information at a particular time, can be used to assess the patient's decision‐making capacity at each individual decision point in their treatment.^174^

### 6.1 Pregnancy, IDD, and epilepsy

Women with IDD contemplating pregnancy face the same considerations as women with epilepsy in general.^175^ Hormonal factors, effective contraception, and an awareness of potential teratogenicity (structural, developmental, and neurodevelopmental) should be incorporated into the overall management from the moment an ASM is initiated.^176^ The same ASMs are considered appropriate in pregnancy among women with epilepsy and IDD as for women with epilepsy in general, but how information, consent, and adherence are managed will need to be handled differently. Counseling before conception will need to discuss not only genetic aspects and the risk of passing the condition on but also the individual's ability to look after her condition during pregnancy and the baby after delivery, as well as the need for support to ensure the safety of the mother and the baby. In a review article on sexual and reproductive healthcare for women with IDD, but not specifically epilepsy, practice recommendations were provided to guide primary care clinicians in managing the many different aspects involved.^177^ A population‐based study on pregnancy and birth outcomes in Sweden in women with IDD showed that a higher proportion of women with IDD were teenagers, obese, smoked more often, and were single compared with women without IDD.^178^ Among these, a medical history of epilepsy at the first antenatal visit was registered 14 times more often for women with IDD (5.5%) than for women without IDD (0.4%). Women with IDD had more often a preterm birth, cesarean section, non‐use of nitrous oxide, and discharge from hospital to a place other than home. The study concluded that pregnant women with IDD should be considered a risk group, requiring better tailored pre‐ and intrapartum care and support.^178^

## 7 GASTROINTESTINAL ISSUES

The dual relationship between the gut and the brain is reinforced by evidence that patients with epilepsy are reportedly more likely to have functional gastrointestinal disorders compared with healthy individuals.^7^ These disorders include irritable bowel syndrome, functional constipation, and functional dyspepsia.^7^, ^179^ The reason for this derives from the interplay between the enteric system, the vagal nerve, the immune and neuroendocrine systems, as well as substances produced by the gut‐residing microbiota, an increasingly studied area within epilepsy.^180^, ^181^, ^182^ Furthermore, patients with inflammatory bowel diseases may have a higher chance of developing seizures than those without.^183^ There are many gastrointestinal disorders in people with IDD and epilepsy, but they are generally not well studied.^7^ Carers of people with IDD and epilepsy often report constipation as a trigger for seizures.^184^ Additionally, both constipation and diarrhea have been reported as side effects of ASMs.^7^ Drooling is a significant problem in individuals with physical comorbidities, and seizure frequency needs to be monitored if anticholinergic agents are used in view of the potential for seizure exacerbation.^185^ Botulin toxin injections for sialorrhea may be considered.^186^ Where there is a percutaneous endoscopic gastrostomy (PEG), adherence to medication can continue during intercurrent illness, but this can be associated with its own complications.^187^ There may be interactions between ASMs and medications frequently used for gastrointestinal disorders, such as proton pump inhibitors.^188^ When managing patients with combined epilepsy and IDD, clinicians need to review how gastrointestinal disease, epileptic seizures, and the drugs used to treat each condition influence one another and adjust ASM treatment accordingly.

### 7.1 Dietary treatments

There are certain nutritionally responsive epilepsies, often associated with IDD, that require specific management within metabolic services, such as GLUT1 deficiency.^189^ Others include pyruvate dehydrogenase complex deficiency (PDCD^190^), succinic semialdehyde dehydrogenase (SSADH^191^) deficiency, non‐ketotic hyperglycinemia (NKH), creatine deficiency syndromes, and vitamin‐responsive epilepsies. Epilepsy and IDD are common among individuals with GLUT1 deficiency, with one study reporting epilepsy in 83% and IDD in 50% of affected individuals.^189^ Treatment of epilepsy in GLUT1 deficiency is the same as for those without IDD, with the ketogenic diet as first‐line treatment,^192^ with additional support as discussed below.

More generally, outside of metabolic disorders, dietary therapies, including ketogenic approaches, remain a potential option for drug‐resistant epilepsy but require careful consideration in adults with IDD.^193^, ^194^, ^195^, ^196^, ^197^ While such treatments may be beneficial in selected individuals, their use is often limited by practical and clinical challenges. Strict dietary adherence may be difficult to achieve due to feeding problems,^28^, ^198^ sensory sensitivities, behavioral rigidity,^113^ and reduced capacity to understand or participate in the dietary plan. Gastrointestinal comorbidities such as constipation, gastroesophageal reflux, or slowed gastric emptying^7^, ^193^, ^194^, ^199^ may further complicate tolerance and increase the risk of adverse events, including dehydration and metabolic instability.

The successful implementation of ketogenic diets in this population typically requires multidisciplinary support from epilepsy specialists, dietitians, metabolic teams, caregivers, and residential staff. Monitoring must be rigorous and regular, considering the person's weight, hydration status, bowel habits, and biochemical parameters.^200^ Caregiver burden is often substantial, and the overall benefit–risk profile must be discussed in detail. Patient characteristics that could guide such benefit–risk considerations include characteristics associated with the epilepsy profile (seizure burden, drug resistance, underlying cause, other treatment options), the patient's medical and nutritional status (including comorbidities such as metabolic disorders incompatible with ketosis), as well as cognitive ability and behavioral‐related factors, and consideration of the family or caregiving setting. The ILAE provides recommendations and a useful resource for the pre‐assessment of patients for ketogenic diet therapy for drug‐resistant epilepsy.^201^ Dietary therapies should be reserved for individuals in whom potential benefits outweigh the practical and medical constraints, and for whom appropriate support can be reliably guaranteed.

## 8 VACCINATION

In general, vaccination is recommended, where indicated, for individuals with epilepsy,^202^, ^203^ and should only be postponed in cases of moderate to severe acute illness to avoid overlapping of vaccine adverse effects with symptoms of the underlying disease. Indeed, a lack of capacity to consent should not prevent access to public health interventions. While vaccination may trigger febrile seizures in some DEEs,^204^ such as Dravet syndrome, it does not alter disease progression.^205^ Clinicians should in general encourage vaccination in patients with DEEs, as it helps prevent severe febrile infections.^206^ In DEEs with febrile‐induced seizures, prophylactic antipyretics may be recommended to prevent fever.^207^ Prophylactic measures with preventive antipyretic medication, for example, are recommended in Dravet syndrome due to the increased risk of prolonged seizures with fever.^206^ In specific cases with seizures triggered by previous vaccines, subsequent vaccinations should be administered under medical supervision with prophylactic antipyretics, and inpatient revaccination protocols may be used if available.

## 9 PERSONALIZED MEDICINE

Personalized medicine in the management of epilepsy and IDD is increasingly recognized as a crucial approach to optimizing therapeutic outcomes and improving QoL.^208^ Treatment selection must consider the heterogeneity of these conditions, considering genetic, clinical, and environmental factors that influence both seizure control and overall wellbeing.^209^, ^210^ Advances in genetic testing and biomarker identification have significantly contributed to the ability to tailor treatments in some conditions to individual patients. For example, the diagnosis of Dravet syndrome, often due to pathogenic variants in the sodium channel, voltage‐gated, type I, alpha subunit (*SCN1A*), leads to the avoidance of sodium channel blockers, more options for treatment, and may lead, in time, to targeted therapeutic strategies. Novel therapeutic approaches include antisense oligonucleotides (STK‐001), adenovirus‐mediated upregulation of transcription of *SCN1A* (ETX101), and CRISPR/Cas9 technology, among others.^211^

The integration of pharmacogenomics into clinical practice may allow for better prediction of drug efficacy and adverse effects, reducing the trial‐and‐error approach that has traditionally characterized epilepsy treatment. Pharmacogenomics can reduce medication side effects by, for example, avoiding specific medications in people predisposed to allergic reactions,^212^ and implementing different titration regimens in those who are slow metabolizers, for example, of brivaracetam or clobazam.^213^ A recent consensus on rare epilepsies highlighted the importance of multidisciplinary care and early implementation of targeted therapies and non‐pharmacological interventions to optimize long‐term outcomes, especially during the transition to adulthood.^208^ In Italy, a survey assessing the prevalence of single‐gene etiologies for DEEs revealed a significant increase in molecular diagnoses over the past decade, highlighting the critical role of genetic screening in guiding precision medicine strategies.^209^

In addition to genetic considerations, the clinical phenotype plays a pivotal role in guiding therapy. Factors such as seizure type, frequency, severity, and associated comorbidities, including cognitive and behavioral difficulties, must be considered when selecting treatment options.^214^ The choice of ASMs is influenced not only by seizure characteristics and epilepsy syndrome but also by the potential impact on cognitive function, behavior, and sleep.^215^ As discussed above, personalized approaches aim to balance seizure control with QoL, minimizing side effects such as sedation or cognitive impairment. CBD, fenfluramine, and stiripentol have emerged as a promising adjunctive therapy for severe epilepsy in Dravet and Lennox–Gastaut syndromes.^216^, ^217^, ^218^, ^219^, ^220^, ^221^ CBD and everolimus have also shown efficacy in seizures associated with tuberous sclerosis complex (TSC).^222^, ^223^ These examples further underscore the importance of targeted treatment approaches.^214^ Personalization also extends to individualized rescue treatment protocols, as discussed above.

Despite advances, challenges remain in the widespread implementation of personalized medicine in DEEs as well as IDD in general. Limited access to genetic testing, variability in treatment response, and economic constraints pose significant barriers.^224^ Addressing these challenges requires a multidisciplinary approach that includes education, advocacy, and policy initiatives to ensure equitable access.^208^ Moving forward, efforts should focus on refining personalized treatment algorithms and integrating emerging technologies to further enhance individualized care strategies.

## 10 CONCLUSION

Effective management of adults with IDD and epilepsy requires a comprehensive, person‐centered, multidisciplinary approach to address the medical, cognitive, behavioral, and social dimensions (Figure 1). Uncontrolled seizures, polypharmacy, comorbidities, and behavioral challenges significantly affect the QoL of the individual and carers/family, imposing considerable social and economic burdens on patients, caregivers, and society. Implementing multidisciplinary team‐based care is critical, involving neurologists, psychiatrists, psychologists, therapists, social‐care workers, and dedicated nursing support.^208^ Such teams need to create personalized care plans, ensuring regular reviews and proactive collaboration with primary care physicians and caregivers to keep the patient's needs central (Figure 2). A protocol example has been proposed for establishing such multidisciplinary teams in TSC,^225^ which includes the constitution of a core team of specialists guiding and coordinating the management for these patients with all members of the team and the caregivers. Acknowledging the complex interplay between epilepsy and IDD, particularly during critical life transitions, is essential for improving patient outcomes. Despite advancements, substantial gaps in equitable access to integrated care and tailored support services persist globally. Addressing these disparities through targeted research, guidelines, improved educational resources, and equitable resource allocation remains an urgent priority for enhancing QoL and reducing healthcare inequalities in this vulnerable population.

## CONFLICT OF INTEREST STATEMENT

Elena Fonseca: Consulting honoraria from UCB and Jazz Pharmaceuticals, and speaker fees, research funding, and travel support from UCB, Esteve, Eisai Inc., BIAL, Jazz Pharmaceuticals, Angelini Pharma, and Sanofi Genzyme. Antonella Riva: Served on scientific advisory boards for Biocodex, UCB, Jazz Pharmaceuticals, and PTC Therapeutics, and received travel grants from Jazz Pharmaceuticals. Samuel López‐Maza: Travel support from Eisai Inc., Angelini Pharma, and Jazz Pharmaceuticals. Malin Zaddach: Fees for travel expenses, courses, and conference registration from Jazz Pharmaceuticals. Marie‐Claire Porter: Nothing to disclose. Ingo Borggraefe: Scientific grants from Roche; honoraria for speaker and advisory role from Desitin, Eisai, Jazz Pharmaceuticals, Neuraxpharm, UCB, Ethypharm, Stoke Pharmaceutics; member of data safety monitoring board for Praxis Pharma. Lance Watkins: Honoraria for speaker and educational work from Veriton Pharma and UCB, outside of this project. Andreas Schulze‐Bonhage: Received research support from DFG, EU, German Ministry of Science and NIH, as well as from Precisis and UNEEG; received personal honoraria for lectures or advice from Angelini Pharma, BIAL, Livanova, Precisis, UCB, and UNEEG. Rohit Shankar: There is no direct disclosure or conflict of interest for this submitted body of work. RS developed the non‐commercial and free to use SUDEP and Seizure Safety Checklist and the EpSMon app to reduce the risk of SUDEP and enhance seizure safety. RS is the Chief Investigator of the NIHR adopted national Ep‐ID register. The Register is supported and monitored by the National Institute of Health Research UK. The funding for each molecule examined by the Register is via an Investigator Initiated Support grant from each of the molecule's parent company. The funding is to RS's NHS institution and goes toward the salary of the research coordinator and the institution's project oversight costs. The contributing companies till date include Eisai, UCB, Bial, Jazz Pharmaceuticals (previously GW Pharmaceuticals), and Angelini. This work sits outside the submitted work. In addition to the above, RS has received institutional research, travel support, and/or honorarium for talks and expert advisory boards from LivaNova, UCB, Eisai, Neuraxpharm, Veriton Pharma, Bial, Angelini, UnEEG, and GW/Jazz Pharmaceuticals outside the submitted work. He holds or has held competitive grants from various national grant bodies including Innovate, Economic and Social Research Council (ESRC), Engineering and Physical Sciences Research Council (ESPRC), National Institute of Health Research (NIHR), NHS Small Business Research Initiative (SBRI), and other funding bodies, including charities, all outside this work. Pasquale Striano: Fees for advisory board participation, lectures, and research support from UCB, Zogenix, GW/Jazz Pharmaceuticals, Eisai, Biocodex, and Angelini Pharma. Lina Nashef: Fees for advisory board participation, lectures, and research support from Jazz Pharmaceuticals, UCB, and Angelini Pharma.

**Test yourself**

1. What is a key challenge in diagnosing epilepsy in adults with intellectual development disorders?
  - Accurate seizure diaries
  - Access to imaging
  - Communication barriers
  - Availability of neurologists
2. Which of the following is correct regarding quality of life (QoL) evaluation in people with intellectual development disorders (IDD) and epilepsy?
  - QoL priorities of people with IDD and epilepsy need to be better defined
  - Seizure freedom is the only determining factor for a better QoL in this group
  - Polypharmacy and antiseizure medication side effects do not influence QoL
  - Available instruments can be used in this population, as in other people with epilepsy
3. Transition from pediatric to adult care is most hindered by:
  - Pediatric care being less specialized
  - Adult neurologists have less expertise
  - Disruption of continuity of care
  - Reduction of seizures at transition
4. Which aspects should be considered when evaluating sleep quality in people with intellectual development disorders and epilepsy?
  - Insomnia, arousals, and daytime sleepiness
  - Nocturnal seizures
  - Medications that could disrupt or benefit sleep quality
  - All of the above
5. What is correct regarding epilepsy in women with intellectual development disorders (IDD) and epilepsy?
  - The combined contraceptive pill is the only option for managing catamenial exacerbation of seizures
  - Potential interaction of hormonal therapies and some antiseizure medications need to be considered
  - All hormonal therapies are contraindicated in those on lamotrigine
  - Bone health need not be considered in relation to contraception in people with IDD and epilepsy
6. What is false regarding vaccinations in people with intellectual development disorders (IDD) and epilepsy?
  - Vaccination is generally, where indicated, recommended for people with IDD and epilepsy
  - In patients with febrile‐induced seizures, prophylactic antipyretics may be recommended before a vaccine is administered
  - In some syndromes with febrile seizures, vaccinations alter disease progression in the long term, so they should be avoided
  - If there is a history of previous vaccination‐induced seizures, subsequent vaccinations should be administered under medical supervision with prophylactic antipyretics
7. What is a key challenge in the medical treatment of epilepsy for people with intellectual development disorders?
  - Limited self‐reporting of adverse events, seizure semiology, and frequency
  - The effect of polytherapy on psychological and cognitive function
  - Misdiagnosis of non‐epileptic seizures as epilepsy, which may lead to overmedication
  - All of the above
8. Adaptations of service provision for people with intellectual development disorders (IDD) and epilepsy may include all but one of the following:
  - Availability of resources close to community‐based services
  - Providing sufficient time for outpatient visits, adapted to the needs of the individual
  - Allowing carers to stay overnight during hospital admissions
  - Segregating people with IDD and epilepsy from community‐based healthcare resources, since they can only be managed at specialized epilepsy units
9. People with intellectual development disorders and epilepsy are at higher risk of:
  - Fractures
  - Higher mortality rates
  - Sudden death in epilepsy
  - All of the above
10. Appropriate management of cognitive and behavioral comorbidities of people with intellectual development disorders and epilepsy includes:
  - Focusing on neuroleptics as the only treatment strategy for controlling these symptoms
  - Choosing an antiseizure medication regimen for seizure control without concern for psychiatric side effects
  - Reducing polypharmacy and total drug load with careful titration
  - Prioritizing seizure management since cognitive and behavioral symptoms cannot be improved

*Answers may be found in the* *Supporting information*.

## Supporting information


Data S1.

## Data Availability

Data sharing not applicable to this article as no datasets were generated or analysed during the current study.

## APPENDIX A CLINICAL CASE STUDIES

**Case 1**

A 21‐year‐old woman with cyclin‐dependent kinase‐like 5 (CDKL5)‐associated developmental and epileptic encephalopathy (DEE) has had a severe neurodevelopmental disorder and drug‐resistant epilepsy since the age of 2 months with myoclonic, tonic, and multifocal seizures. She currently has intellectual development disorders, microcephaly, tetraparesis, dysphagia (she feeds through a gastrostomy), bisphincteric incontinence, chronic constipation, alteration of sleep–wake cycle, and behavior disorder. Her treatment when she was first evaluated in the epilepsy clinic after transition to adult care included phenobarbital, rufinamide, vigabatrin, and risperidone. A recently initiated ketogenic diet resulted in a slight reduction in seizure frequency.

However, shortly after the transition visit, she presented an emergency on several occasions with respiratory failure secondary to recurrent aspiration and required non‐invasive ventilatory support on one occasion. She developed significant behavioral disturbance during admission, leading to an increase in doses of neuroleptics, which worsened her alertness and cognitive state. Her seizures became more frequent and severe during admission.

Following evaluation by the nutrition and physical therapy support teams, slowed gastric emptying was identified, which along with the presence of ketones resulted in nausea and vomiting. After a multidisciplinary discussion, a progressive transition to a normal diet was carried out with significant improvement and no further episodes of aspiration. There was no significant increase in seizure frequency. Her alertness and behavioral symptoms also improved, allowing for de‐escalation of neuroleptic doses.

This case illustrates the need for a multidisciplinary assessment to address the complexity in this case. Non‐pharmacological approaches may be necessary to improve not only seizures but also nutrition and behavior, and reduce morbidity in similar cases.

**Case 2**

A 23‐year‐old man with generalized epilepsy of unknown etiology presented aged 2 years. Brain imaging, lumbar puncture, and genetic testing were not diagnostic. He has intellectual development disorders with speech and language delay. In childhood, he experienced tonic, atonic, absence, and generalized tonic–clonic seizures (GTCS) in addition to non‐convulsive status. GTCS became rare following VNS. In the adult clinic, his main presentation was of prolonged non‐convulsive status, a state that fluctuated in severity. Accurate seizure counts of absences/non‐convulsive status were not possible. His family reported discrete, frequent daily events when he ‘zoned out’ for variable periods and prolonged periods when he was less interactive, such that his parents found it difficult to feed and hydrate him. EEG showed almost constant generalized epileptiform discharges. Medications tried with some benefit included revisiting valproate, ethosuximide, topiramate, perampanel, and clobazam, but tolerance limited doses and benefit.

Within the framework of a Lennox–Gastaut‐like presentation, he was started on cannabidiol (CBD, Epidyolex® [EU]/Epidiolex® [US]) as add‐on treatment to valproate (valproate was later reduced to 400 mg twice daily due to liver enzyme rises which settled), clobazam 5 mg AM and 10 mg PM. His parents had also reduced his topiramate to 15 mg, only giving it to him every 3 days, which they felt was the maximum he could tolerate. When last seen, the CBD dose had been titrated to 400 mg twice daily (weight ~68 kg). The addition of CBD resulted in a response which his parents felt was ‘fantastic’: ‘zoning out’ episodes became infrequent; he was eating and drinking normally; they reported a ‘reawakening’; he had become much more engaged and was learning new words. He was more steady walking, including up and down stairs. His behavior was reportedly good, though some repetitive behavior was more evident. Repeat EEG still showed generalized discharges, less frequently than before. Further titration of his medication is planned. This case illustrates a clear benefit associated with ASMs, in this case CBD, with reduced epileptiform activity on EEG, better cognition, daily function and behavior, with improved QoL for the individual and his family.
