# Supplementary material for: Holistic management of epilepsy in adults with intellectual development disorders
Source: Epileptic Disord. 2026 May 22;28(4):981–1006. doi: 10.1002/epd2.70272 (PMC13499271; doi:10.1002/epd2.70272)
Supplement: Supplementary file 1 [file EPD2-28-981-s001.docx]

**Answers**

1. Correct answer: C

2. Correct answer: A

3. Correct answer: C

4. Correct answer: D

5. Correct answer: B

6. Correct answer: C

7. Correct answer: D

8. Correct answer: D

9. Correct answer: D

10. Correct answer: C
